# Multiplex PCR point of care testing versus routine, laboratory-based testing in the treatment of adults with respiratory tract infections: a quasi-randomised study assessing impact on length of stay and antimicrobial use

**DOI:** 10.1186/s12879-017-2784-z

**Published:** 2017-10-10

**Authors:** Denise Andrews, Yumela Chetty, Ben S. Cooper, Manjinder Virk, Stephen K Glass, Andrew Letters, Philip A. Kelly, Malur Sudhanva, Dakshika Jeyaratnam

**Affiliations:** 1 Division of Acute Medicine, King’s College Hospital NHS Foundation Trust, London, SE5 9RS UK; 2 Mahidol-Oxford Tropical Medicine Research Unit, Faculty of Tropical Medicine, Mahidol University, Bangkok, 10400 Thailand; 3 Centre for Tropical Medicine and Global Health, Nuffield Department of Clinical Medicine, University of Oxford, Oxford, UK; 4 South London Specialist Virology Centre, King’s College Hospital NHS Foundation Trust, London, SE5 9RS UK; 5 Department of Medical Microbiology, Cheyne Wing, King’s College Hospital NHS Foundation Trust, Denmark Hill, London, SE5 9RS UK; 6 Infection, Prevention and Control Team, King’s College Hospital NHS Foundation Trust, London, SE5 9RS UK

**Keywords:** Point of care, FilmArray®, Respiratory pathogens, Respiratory viruses, Respiratory tract infection, Length of stay, Multiplex PCR, Antimicrobial stewardship, Adults

## Abstract

**Background:**

Laboratory-based **r**espiratory pathogen (RP) results are often available too late to influence clinical decisions such as hospitalisation or antibiotic treatment due to time delay in transport of specimens and testing schedules. Ward-based i.e. point of care (POC) testing providing rapid results may alter the clinical management pathway.

**Methods:**

FilmArray® RP polymerase chain reaction (PCR) systems were placed in three in-patient and out-patient medical areas. Patients presenting with influenza-like illness /upper respiratory tract infection +/− lower RTI were recruited between January–July 2015. FilmArray® POC testing occurred on even days of the month (intervention) or routine, laboratory-based RP PCR testing +/− atypical serology on odd days (control). The primary outcome was length of hospital stay. The secondary outcomes were impact on the use of antimicrobials, readmissions, all-cause mortality, length of ward stay and turn-around time (TAT) (time to result from admission).

**Results:**

Of 606 eligible patients, 545 (89.9%) were included; 211 in the control arm and 334 in the intervention arm. 20% of control arm patients and 24% of intervention arm patients had an RP detected. POC testing was not associated with the primary outcome measure, length of stay, but reduced the TAT from 39.5 h to 19.0 h, *p* < 0.001. Only the prescribing decision differed between study arms, p < 0.001. When antivirals were given, the intervention was associated with a reduction in the median time to the first dose of 36 h and allowed appropriate treatment of mycoplasma infection.

**Conclusions:**

We found no association between respiratory PCR POC testing and length of stay or most of the secondary outcomes except the antimicrobial prescribing decision. This was probably due to a delay in initiating FilmArray® testing. Despite this, POC testing allowed time-critical antivirals to be given significantly faster, appropriate mycoplasma treatment and results were available considerably faster than routine, laboratory-based testing. Ward-staff of all grades performed POC testing without difficulty suggesting potential use across many divergent healthcare settings. Further studies evaluating the implementation of rapid respiratory PCR POC testing and the effect on length of stay and antimicrobial use are required.

**Trial registration:**

ISRCTN10470967, Retrospectively Registered, 30/6/2015.

## Background

Respiratory tract infections (RTI) place a significant burden on health systems globally, particularly during the annual respiratory season epidemics [1]. Diagnostic tests for respiratory pathogens (RP) are usually laboratory–based with an inherent delay in time to result relating to specimen in transit to the laboratory and laboratory testing schedules, for example once a day, and/or not performed on weekends and holidays. For this reason or due to the nature of the test (culture, serology or batch molecular testing) results are rarely available to the clinician when the patient is first assessed. Consequently, though respiratory viruses are frequently isolated in community acquired pneumonia (CAP) [2] and are reported to be responsible for 12.8% of CAP cases admitted to UK hospitals [3], the decision to manage as a viral RTI or treat for bacterial infection including *Mycoplasma pneumoniae* or *Chlamydia pneumoniae* (*‘*atypical bacteria’) is based upon the clinical scenario and severity criteria such as the CURB-65 score. Thus a proportion of infections will be inappropriately managed with antibiotics or the result may arrive too late for influenza treatment to be effective [4]. The World Health Organization (WHO) states that antimicrobial resistance threatens the effective prevention and treatment of an ever-increasing range of infections [5]. The Centre for Evidence Based Medicine highlights the considerable number of new diagnostic technologies in development to underpin the rational prescribing of antibiotics [6], which extends to antivirals.

Point of care (POC) tests eliminate the need for specimen transportation to the testing laboratory and can be performed on demand by ward staff. By providing faster results, POC tests may influence early treatment decisions such as hospital admission and allow earlier discharge, targeted antimicrobial prescriptions and better antimicrobial stewardship. POC testing should also reduce cross-transmission and subsequent nosocomial outbreaks related to viral RTI cases that are undiagnosed and patients not placed in appropriate isolation. However, current POC tests for respiratory viruses are generally antigen detection tests, usually only detect influenza and respiratory syncytial virus (RSV) and their sensitivity can be suboptimal [7, 8]. The BioFire FilmArray® respiratory panel (BioFire Diagnostics, Salt Lake City, UT, a bioMerieux Company) detects 14 respiratory viruses: influenza virus types A and B (with influenza A subtyping), adenovirus, coronaviruses HKU1, NL63, 229E and OC43, human metapneumovirus, human rhinovirus/enterovirus, parainfluenza virus types 1–4 and RSV and 3 bacteria: *Mycoplasma pneumoniae, Chlamydia pneumoniae* and *Bordetella pertussis*. FilmArray® lends itself to POC testing as it is a small, desktop, fully-automated nested multiplex PCR in an enclosed disposable pouch requiring only 2 min of hands-on time with results available in about 1 hour [9]. Though more expensive than single or low multiplex laboratory or POC tests, clinical outcomes such as a reduced length of stay and reduction in inappropriate antimicrobial usage resulting from the rapid and extended panel may offset test costs. Thus understanding FilmArray®‘s clinical utility as a POC test early in a patient’s admission is important.

We undertook a study to assess the FilmArray® RP panel as a POC test compared to routine, laboratory-based detection methods in order to assess the impact on length of stay and antibiotic utilization. It is the first study, to our knowledge, in which ward-staff were performing the FilmArray® RP panel as a POC test.

## Methods

### Study aims

The aim of the study was to determine whether in adults presenting with upper respiratory tract infection (URTI)/influenza-like illness (ILI) +/− lower respiratory tract infection (LRTI), FilmArray® RP panel POC testing, when compared to the routine, laboratory-based RP testing was associated with length of hospital stay or antimicrobial use.

### Study setting

The study took place in a 900 bedded teaching and tertiary referral site of a two-site 1450 bedded acute NHS hospital Trust in London.Four FilmArray® systems were placed in side-rooms across three adult (>16 years) wards: two Acute Medical Units (AMUs) and the Medical Assessment Centre (MAC). The AMUs are 28 and 30 bedded short-stay, acute medical wards to which patients are admitted from the Emergency Department (ED). The MAC is an area to which out-patients can be referred for review by clinicians. It is open daily from 8 am to 10 pm. MAC patients are assessed and then admitted to an AMU or discharged.

### Study design

A quasi-randomised trial design was used such that patients were enrolled in to the control arm on odd days of the month and in to the intervention arm on even days of the month. This was the most pragmatic design that could be implemented on the study wards. The study was not blinded.

Study-ward staff were educated about the study and consent taking, given a staff information sheet and trained to use the FilmArray® respiratory panel as per manufacturer’s instructions. Competency assessments were conducted. All staff were required to don personal protective equipment when performing each assay. FilmArray® tests were to be ordered and performed by study-ward staff, however if they were unable to do the testing e.g. due to clinical duties, the study investigators, who worked on weekdays until 7 pm and a half-day on weekends and bank holidays, performed the FilmArray® test. In order to interpret the FilmArray® results out of hours, study-ward staff consulted a standard operating procedure describing each pathogen, the type of diseases associated with it, the groups at risk of severe infection, any medical management and the infection control precautions required, if at all. They also had direct contact numbers for the study investigators who were available at all hours. Eligible patients were identified by ward staff or study staff. Written informed consent was obtained by ward staff before patient participation.

In the control arm, combined nose and throat flocculated swabs (Copan Diagnostics, Italy), were placed in viral transport medium (VTM, Copan) which was transported to the laboratory by hospital porters as is routine. The standard, routine diagnostic assays for viral pathogens used in the control arm are in-house developed real-time PCRs with 4 separate multiplex assays (influenza A (H1N1) pdm09 matrix gene RNA^1^ and H1 RNA, influenza A virus RNA, influenza B virus RNA, rhinovirus RNA, RSV subgroup A and subgroup B RNA, parainfluenza viruses 1, 2 and 3 RNA and human metapneumovirus RNA) and an adenovirus monoplex [10–12]. Outside this study, there is no rapid/POC RP testing at this hospital. If requested by the clinical team, *Mycoplasma pneumoniae* and *Chlamydia pneumoniae* were tested for in the control arm using the laboratory’s routine, complement fixation tests (CFT) (Launch Diagnostics, Kent, UK and TCS Biosciences, Buckingham, UK). A comparison of the pathogens detected by the FilmArray® and the routine diagnostic tests is shown in Table 1. The routine tests were performed on site by qualified Health Care Scientists. PCR results were available at around 4 PM every weekday and on weekends. Routine serology testing was batch-tested once per week.

**Table 1:** FilmArray® panel compared to the routine laboratory-based PCR and non-PCR methods

	Control		Intervention
Pathogen	Routine PCR	Serology/culture	FilmArray®
Adenovirus	✓	–	✓
Coronavirus HKU1, NL63, 229E, OC43	Not tested	–	✓
Human metapneumovirus	✓	–	✓
Human rhinovirus/ enterovirus	✓	–	✓
Influenza A virus	✓	–	✓
Influenza A virus H1	✓	–	✓
Influenza A virus H3	✓	–	✓
Influenza A virus H1–2009	✓	–	✓
Influenza B virus	✓	–	✓
Parainfluenza virus 1,2,3	✓	–	✓
Parainfluenza virus 4	Not tested	–	✓
Respiratory syncytial virus	Subgroups A/B	–	✓
*Bordetella pertussis*	Not tested	Culture	✓
*Chlamydia pneumoniae*	Not tested	Complement Fixation test (CFT)	✓
*Mycoplasma pneumoniae*	Not tested	Complement Fixation test (CFT)	✓

In the intervention arm, combined nose and throat flocculated swabs (Copan Diagnostics, Italy), were placed in viral transport medium (VTM, Copan) and thereafter a rehydration buffer and 200 μl of the VTM was introduced into the FilmArray® pouch by ward-based staff (POC testing) on even days of the month (intervention arm). FilmArray® was validated against the routine method to ensure satisfactory performance characteristics but a head to head comparison was not included as part of this study. External (Quality Control for Molecular Diagnostics [QCMD], Glasgow, UK) and internal quality control specimens were tested by FilmArray® during the study. The use of the FilmArray® was approved by the Trust Point of Care Testing Committee.

All results were uploaded to the hospital results reporting system. Positive results for both study arms were telephoned by a microbiologist or virologist to the health care provider. Antimicrobial stewardship activities did not change during the study.

All other diagnostic specimens e.g. for bacterial culture were sent to the laboratory as usual for both study arms. These results were not included in the analysis.

### Eligible patients

The inclusion criteria were that patients were ≥16 years of age, with mental capacity to give written informed consent and presenting with URTI/ILI defined as symptoms including fever or feeling feverish (chills), cough, sore throat, runny or stuffy nose, muscle-aches or body-aches, headaches, fatigue (tiredness) and possibly vomiting or diarrhoea [13] +/− LRTI. Patients who did not meet inclusion criteria or with evidence or suspicion of bacterial infection affecting sites other than the respiratory tract were excluded from the opportunity to participate in the study. Patients who requested withdrawal from the study were excluded from the analysis.

### Primary and secondary outcomes

The primary outcome studied was the length of hospital stay defined as the time between hospital admission and hospital discharge. The FilmArray® RP panel is relatively expensive (£89–£133 for the consumables and £28, 000-£33,000 for the capital purchase of the system depending upon the country) compared to currently employed routine tests, (£50 per test for our standard laboratory method). However the average cost for managing pneumonia in the community is estimated at £100 per episode compared with £1700–5100 for hospitalised patients in the UK [14] and $8000 in the US [15]. Thus if a FilmArray® POC result allows earlier discharge, the consequent financial savings from the shorter hospital stay may offset the extra test costs and prove cost-beneficial, hence our choice of primary outcome. The secondary outcomes were antimicrobial prescription/s (antibiotics: any versus none, duration, time to prescription within the first 72 h of the patient’s stay and prescribing decisions within 24 h after the the diagnostic results under investigation: start, stop, de-escalation, escalation and continued use, as assessed by the Chief Investigator), readmission rates and all-cause mortality (both within 30 days of the test) and length of study-ward stay (i.e. removing the length of stay on subsequent wards to which the patient was transferred, where relevant). Escalation was defined as the addition of an antibiotic/s to the existing antibiotic therapy or the substitution of the current agent with a broader agent. De-escalation was the cessation of ≥1 antibiotic when >1 antibiotic was prescribed or the substitution with a narrower agent. We collected these data prospectively and retrospectively from the electronic patient record (EPR) and the electronic patient medicines administration system (EPMA). In some cases, enrolment of the patient into the hospital administrative system occurred after examination and initiation of antibiotic therapy by the physician on the ward or in the ED. Therefore, a negative time to antibiotic administration on the ward was corrected to zero hours to antibiotics. We defined the turn-around time (TAT) of the tests as the time between hospital admission and the time of the result on the FilmArray® system (intervention) or the time of the result on the EPR (control).

For each patient, we collected demographic data, Charlson co-morbidity score, and at the time of admission, CURB-65 score for patients with a diagnosis of community-acquired pneumonia, an early warning score (EWS) [16], peripheral white cell count (WCC), C-reactive protein (CRP) and the day of the week of admission. The type of LRTI e.g. infective exacerbation of chronic obstructive pulmonary disease (COPD), bronchitis, pneumonia was not recorded.

### Statistical methods

The power calculation was based upon length of stay, which had previously been observed to be 3.2 days on the study wards. Rapid pathogen identification has been associated with a reduction of mean hospital length of stay, in one study from 11.9 days to 9.3 days (approx. 20%) (*p* = 0.1) [17]. For a 20% reduction in length of stay (LOS) in the intervention arm, sample size calculations indicated that sample sizes of 1131 cases in each arm were required to achieve 90% power to detect a difference of 0.6 between the null hypothesis that both group means are 3.2 days (76.8 h, based upon data collected from the study wards) and the alternative hypothesis that the mean of group 2 is 2.6 days (62.4 h) with known group standard deviations of 5.2 and 5.2 and with a significance level (alpha) of 0.05.

The primary outcome was analysed with a linear regression model (after log-transformation of length of stay data) according to a pre-specified analysis plan with an individual patient taken as the unit of analysis. Secondary outcomes were analysed using linear regression models for continuous outcome data, logistic regression for binary outcomes, and negative binomial regression for count outcome data. For all these patient-related outcomes we adjusted for multiple pre-specified potential confounders (age, sex, Charlson score, EWS, WCC, CRP). Pearson’s Chi-squared test was used to test for differences between the arms in categorical antibiotic prescribing decisions with *p*-values calculated by 10,000 Monte Carlo replicates (to avoid problems associated with small cell counts associated with the usual asymptotic p-values). A t-test was used to compare the time to test between the two arms. A planned subgroup analysis was performed as above for primary and secondary outcomes excluding patients who had infection proven elsewhere after enrolment as it is plausible that a respiratory pathogen POC result would not alter LOS or antibiotic use when the patient had another infective diagnosis made. Analysis was conducted in *R* [18]. Multiple imputation was used to account for missing data using the package *mice* [19].

## Results

**Fig. 1:**
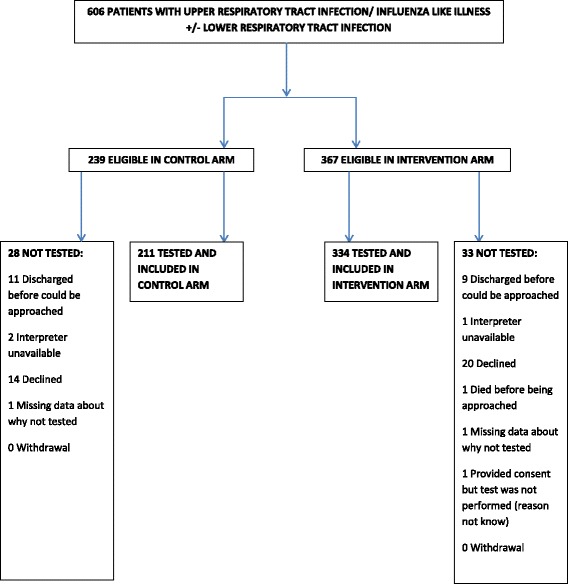
Flow of study participants through the trial. N.B. The total number of patients screened for eligibility was not collected

The study ran from 5th January 2015 until 1st July 2015 as planned. No changes were made to the study protocol. During this time 606 patients met eligibility criteria (Fig. 1). Sixty-one (10.1%) of these patients were not included (33 in the intervention arm, 28 in the control arm) because 20 were discharged before enrolment, an interpreter was unavailable for three, 34 declined participation, one patient died before being approached, one patient consented but the test was not performed and information is missing for two patients. No patients withdrew from the study. Thus 545 (89.9%) patients were enrolled and included in the analysis, 211 in the control arm and 334 in the intervention arm. All statistical analyses were pre-specified; there were no post-hoc analyses.

Baseline characteristics were similar between the two study arms (Table 2). CURB-65 score was missing for 62.9% of patients for whom it was relevant and was omitted from the analysis. One hundred and sixty-five (30%) patients had a negative time to antibiotics changed to 0 h (median − 1.5 h [IQR −3.5 to −0.8]).

**Table 2:** Baseline characteristics

	Control	Intervention
Male gender	52% (110/211)	52% (172/334)
Age (years)^a^	61 (47–73)	67 (47–77)
Charlson score^a^	0 (0–2)	1 (0–3)
White cell count (×10^9^/L)^a^	9.6 (6.8–13.7)	9.6 (6.9–13.1)
C-Reactive protein (mg/L)^a^	38.3 (9.4–123.1)	39.5 (15.4–124.5)
Early warning score^a^	2 (1–4)	2 (1–4)
Admitted on the weekend	22% (47/211)	22% (73/334)

^a^median (IQR), Early Warning Score: Six physiological parameters routinely recorded: i) respiratory rate, ii) oxygen saturations, iii) temperature, iv) systolic blood pressure, v) pulse rate and vi) level of consciousness. In addition, a weighting score of 2 should be added for any patient requiring supplemental oxygen (oxygen delivery by mask or nasal cannulae)

The median time to result from admission was substantially shorter in the intervention arm compared to the control arm (control arm 39.5 h (IQR 25.4–57.6), intervention arm 19.0 h (IQR 8.1–31.7)), two-sample t-test assuming unequal variances, *p* < 0.001. Ward staff of all grades performed 28% of POC tests, 68% of the samples were tested by study investigators and there is no record for 4%. No adverse events were reported.

Overall, 124 (22.8%) of the 545 patients had a positive result (Table 3), 43 (20.4%) in the control arm and 81 (24.3%) in the intervention arm. The viruses and bacteria detected are shown in Table 3. Every virus on the panels was identified except parainfluenza virus type 1, type 2 and type 4. Only single pathogens were detected by routine testing but FilmArray® detected dual infections in five samples. FilmArray® also detected coronaviruses, not detected using standard tests. There were three and four invalid tests in the control and intervention arms respectively, the remaining tests were negative (78.2% control, 74.6% intervention).

**Table 3:** Summary of respiratory pathogen testing results

Result	Routine PCR /serology/culture (Control)	FilmArray® (Intervention)
Total	211	334
Invalid or Inhibitory	3 (1.4)	4 (1.2)
Negative (%)	165 (78.2)	249 (74.6)
Positive (%)	43 (20.4)	81 (24.3)
Positive for a virus (%)	43 (20.4)	76 (22.8)
Positive for a bacterium (%)	0 (0)	5 (1.5)
Influenza A	6	13 (2 dual)
Influenza B	15	18 (1 dual)
Adenovirus	2	3
Parainfluenza virus 1	0	0
Parainfluenza virus 2	0	0
Parainfluenza virus 3	5	8 (1 dual)
Parainfluenza virus 4	Not tested	0
Human metapneumovirus	2	2
Rhinovirus (/enterovirus)	12	16
Respiratory syncytial virus	1	6 (1 dual)
Coronavirus 229E	Not tested	3 (2 dual)
Coronavirus HKU1	Not tested	4 (2 dual)
Coronavirus NL63	Not tested	5
Coronavirus OC43	Not tested	3 (1 dual)
*Mycoplasma pneumonia*	0	5
*Bordetella pertussis*	0	0
*Chlamydia pneumoniae*	0	0

Dual infections: Coronavirus HKU1 & Influenza A, Coronavirus 229E & HKU1, Parainfluenza 3 & Coronavirus 229E, Coronavirus OC43 & RSV and Influenza A & Influenza B

*M. pneumoniae* was the only bacterium on the panels which was identified. Four patients in the control arm had an elevated *Mycoplasma* CFT (1:64, 1:64, 1:32, >1:16). Convalescent serology was not sent, rendering results uninterpretable. All of these results were available after ward discharge, three after hospital discharge, and did not influence management. The TAT was 8–13 days. Five patients in the intervention arm had *M. pneumoniae* detected by FilmArray®. Antibiotics were started for 2 of these cases and extended in 2 after discussion with the Microbiologist.

There was no evidence that the length of hospital stay, the primary endpoint, was reduced by POC testing. The median length of hospital stay was 79.6 h (IQR 41.9–188.9) in the control arm and 98.6 h (IQR 48.1–218.4) in the intervention arm. In the linear model (for log-transformed length of stay data, adjusting for potential confounders) the rapid test was associated with an absolute difference in the natural logarithm of the length of stay of 0.108 (95% CI [−0.089, 0.305]; *p* = 0.28). This corresponds to an 11% (95% CI [−9%, 36%]) increase in length of stay associated with the rapid test arm. Six of 33 patients tested on the MAC in the control arm (18%) and 13 of 67 patients tested in the MAC in the intervention arm (19.4%) were discharged without admission to a hospital ward.

For all but one of the secondary outcomes, there was no evidence that the intervention had an effect (Table 4). Only the prescribing decision within 24 h following the diagnostic results under investigation showed evidence of a difference between study arms (Table 5, *p* < 0.001, Pearson’s Chi-squared test, *p*-value calculated by 10,000 Monte Carlo replicates).

**Table 4:** Summary of secondary outcome measures

Outcome		Control	Intervention	Estimated intervention effect	Adjusted *p* value
Antibiotic use at any time during the hospital stay post-enrolment^a^	Percentage	77% (152/198)	75% (243/324)	aOR (95% CI)1.0 (0.6, 1.5)	0.99
Duration of antibiotic usage (days)^b^	median (IQR)	6.0 (5.0, 7.3)	6.0 (4.0, 7.0)	Absolute difference in natural logarithm of duration (95% CI)-0.08 (−0.22, 0.054)	0.23
Time to antibiotic within the first 72 h of stay (hours)^c^	median (IQR)	0.0 (0.0–3.0)	0.0 (0.0–6.0)	Absolute difference in days (95% CI)2.2 (−1.4,5.8)	0.21
Readmission within 30 days of study participation^a^	Percentage	20% (42/211)	19% (64/333)	aOR (95% CI)0.9 (0.6, 1.4)	0.70
Mortality 30 days post-enrolment^a^	Percentage	4% (9/211)	4% (14/333)	aOR (95% CI)0.9 (0.3, 2.2)	0.79
Length of study ward inpatient stay (hours)^c^	median (IQR)	54 (23, 99)	61 (24, 115)	Absolute difference in natural logarithm of length of stay (95% CI) 0.05 (−0.16, 0.25)	0.66

^a^Based on a logistic regression analysis

^b^Analysed with a linear model (after log transformation)

^c^Analysed with a negative binomial regression

All models adjusted for age, sex, Charlson and Potts scores, day of week, and admission values of WCC and CRP and used multiple imputation to account for missing data. The CURB-65 score was not available for 59% of patients, and was therefore not adjusted for in statistical models. There was less than 5% missing data for all covariates. Missing outcome variables 1) Antibiotics within 72 h and 2) Antibiotics at any time: missing data for 13 patients in the control arm and 10 patients in the intervention arm, 3) Time to antibiotics in the first 72 h: missing data for 1 patient in the intervention arm, 4) duration of antibiotics: missing data for 4 patients in the control arm and 13 in the intervention arm, 5) readmission: missing data for 1 patient in intervention arm, 6) mortality: missing data for 1 patient in intervention arm

**Table 5:** Effect of RP Result on Antibiotic Prescribing

Antibiotic Prescribing Decision Category	Control		Intervention		Total
	number	percent of control arm	number	percent intervention arm	
Continue	128	60.7	165	49.4	293
De-escalate	5	2.4	6	1.8	11
Escalate	4	1.9	26	7.8	30
Start	1	0.5	17	5.1	18
Stop	7	3.3	15	4.5	22
Remain off antibiotics	51	24.1	95	28.4	146
Missing data about decision	15	7.1	10	3.0	25
Total	211		334		545

Control arm de-escalate: 2 stop ≥1 antimicrobial, 3 substitution of Beta-lactam with narrower spectrum Beta-lactam. Intervention arm de-escalate: 2 stop ≥1 antimicrobial, 2 substitution of Beta-lactam with narrower spectrum Beta-lactam, 2 substitution of Beta-lactam with narrower spectrum Beta-lactam and atypical agent was stopped. Control arm escalate: 4 add antibiotic to existing antibiotics (all agents against atypical pneumonia). Intervention arm escalate: 19 add antibiotics to existing antibiotics (14 agents against atypical pneumonia, 5 addition of agents against ‘typical pneumonia’ to atypical agent e.g. Beta-lactam or teicoplanin with ciprofloxacin if penicillin allergic), 7 substitution of Beta-lactam with broader spectrum Beta-lactam

Fifty-one patients had influenza A virus and/or influenza B virus detected by either the routine assay (21) or by the FilmArray® (30). Of these patients, 13 of 21 (62%) in the control arm and 24 of 30 (80%) in the intervention arm were given antivirals. The time to the first dose from the time of admission was known for all but one patient in each arm and was considerably reduced in the intervention arm: median of 60.4 h in the control arm (IQR 22.7–85.2) and 24 h in the intervention arm (IQR 11.6–33.0). Only one patient in each arm was given empiric antivirals but had a no viruses detected.

The planned subgroup analysis excluding patients who had infection proven elsewhere after enrolment e.g. urinary tract infection (*n* = 33) did not substantially alter any of the above results.

## Discussion

We found no evidence for an association between respiratory multiplex PCR (BioFire FilmArray®) POC testing and length of hospital stay when compared to our routine, laboratory-based respiratory PCR and serology testing. There was an association between POC testing and the antibiotic prescribing decision within 24 h after the result. POC testing also produced results considerably faster than laboratory-based assays. We did not investigate whether the POC results actually influenced decision making and the association with the prescribing decision may only reflect that the FilmArray® result was available before the antibiotics were prescribed and not that the prescriber considered the FilmArray® result in their decision making. Similar percentages of patients received antibiotics in both study arms. There were no significant differences for the remaining secondary outcomes between the two study arms.

The hypothesis was that POC testing would reduce the length of stay. However, though the POC test was 1 day faster than laboratory-based testing, the results were available later than anticipated. This was not due to testing performance of FilmArray®, which took only 65 min but related to a delay in the processing of the specimen by the clinical staff on the ward. Sixty-eight percent of POC tests were performed by study investigators reflecting the fact that the study protocol was not initiated by clinical staff as soon as the patient was admitted to the study ward in many cases. Instead testing was delayed until the study investigators visiting the study wards initiated the study protocol. This resulted in a significant delay in time to results. This is in contrast to a trial of MRSA POC screening by our AMU ward staff that had a TAT from admission of 3.7 h [20]. MRSA testing is mandatory and most patients were eligible for inclusion. The delay in the present study may be related to screening for more complex eligibility criteria than the MRSA study and an additional reliance on study investigators present on the ward to perform the test. If this is the case, the TAT would be faster if FilmArray® was embedded as a routine, diagnostic POC test. When ward staff did perform the test, they were from all grades and they performed it without incident. Others report a TAT of 2.3 h for FilmArray® POC testing, though the TAT was from the time of decision to test to the time of result and testing was conducted by trial staff [21]. Thus FilmArray® POC testing can be successfully implemented but this study failed to achieve the optimum TAT.

POC testing was associated with a reduction in time to antivirals for those identified with influenza virus. Antivirals were given a day and a half quicker in the intervention arm and within a day of admission. Given that these drugs are of clinical benefit only if administered within 48 h of symptom onset [4], this is a key, clinical outcome. POC testing allowed changes to therapy for the appropriate treatment of mycoplasma infection; in the control arm, positive results were uninterpretable and were predominantly available after discharge. The ability of FilmArray® to detect coronaviruses allowed for a diagnosis to be made in 15 samples that would have been missed using routine methods. Routine testing only identified single pathogens as opposed to FilmArray® which identified dual infection in 5 patients, of importance for infection control and virus surveillance. Parainfluenza virus types 1, 2 and 4, *Chlamydia pneumoniae* and *Bordetella pertussis* were not detected during the course of this study which did not span the entire winter.

The positive impact on antivirals of the faster time to detection of influenza with FilmArray® was reported by an observational study of paediatric patients, 81% of who were given oseltamivir in a timely manner, which was not possible with the comparator test [22]. An observational study reported a faster time to a negative influenza result with FilmArray® compared to another RT-PCR (46.4 h versus 3.1 h) which shortened unnecessary oseltamivir use by 2 days with an estimated cost saving per patient of $34.16 US [23].

Seventy-five percent of the POC results were negative, providing no information about the aetiology of the infection or the predicted clinical course. Negative results would not be expected to expedite discharge or antibiotic cessation, the main outcomes under consideration here. A paediatric observational study found that patients with a positive respiratory virus PCR result had a 42% shorter duration of intravenous antibiotics [24]. A retrospective study noted a reduced length of stay in children with a positive FilmArray® result reported within 4 h, not seen with negative tests [25]. The reduced time to detection of influenza with laboratory-based FilmArray® was associated with significantly lower odds ratios for admission, length of stay, duration of antibiotics and chest X-rays when compared to positive routine tests in a retrospective study of adults [26]. A randomised trial noted that patients with positive FilmArray® POC results received shorter courses of antibiotics and had shorter hospital stays than those with negative POC results [21]. Our 20–24% positive yield may be because our study was seasonally limited and extended in to the summer months. Further, we did not record the types of RTI, which may have resulted in this lower than expected percentage. Another UK, adult study between September 2012 and February 2014 identified viruses in 30% of patients with lower RTI [27].

The POC result was too slow to influence initial antibiotic decision making as the median time to antibiotics from admission was 0 h. This rapid initiation of antibiotics was also found in a randomised trial of FilmArray® POC testing [21] and is consistent with guidelines that recommend antibiotic treatment for CAP within 4 h of presentation to hospital [3]. Therefore, we would expect almost all patients to be initially started on an antibiotic. However with the delay in POC testing we would not expect this parameter to be impacted, i.e. if testing had been done in an appropriate time frame (<4 h after patient evaluation) the subsequent initiation or discontinuation may have been significantly influenced. Some of this reflects a continuation on the study wards of antibiotics started in the ED. Even with a positive POC result, AMU doctors may be dissuaded on safety grounds from stopping or de-escalating antibiotics that were started on the basis of a clinical assessment that they did not witness in the ED. Our findings are consistent with a randomised trial of FilmArray® POC testing, which found that the mean duration of antibiotics did not differ between the FilmArray® and control arms. However that group identified that a greater proportion of patients in the intervention arm (with a POC result) than in the control arm received only a single dose of antibiotics or <48 h of antibiotics [21], something that we did not assess in the present study. Other trials of RP diagnostics in adults, including FilmArray®, have found that PCR detection of only a viral pathogen coupled with a low procalcitonin level led to antibiotic cessation in only 32% of cases [28] or a trend towards fewer days of antibiotic treatment off-set by only 4/18 patients having their antibiotics stopped [29]. The authors of the latter study advocate real-time stewardship with RP results, which was omitted from the intervention in the present study. Rapid pathogen identification with antimicrobial stewardship has been associated with a significant reduction of hospital costs for adult in-patients [17].

This study has other limitations, nearly all due to limited resources. We employed a pragmatic quasi-randomised design, allocating patients to the intervention arm on even days making the study vulnerable to bias due to differences in patients allocated to the study arms. Though we found no evidence for such differences, and the outcome analysis adjusted for several pre-specified potential confounders, a fully randomised design would have provided a stronger level of evidence. There were more patients in the intervention arm suggesting that the patient recruitment processes in the two arms were not equivalent and this may reflect enthusiasm for the FilmArray®. This may also reflect increased disease severity in the intervention arm and a need to identify the cause of the disease, thereby resulting in a biased use of the FilmArray® in this cohort. CURB-65 scores were not assessed and therefore an accurate comparison of the severity of the pneumonia in the two arms of the study could not be determined. Further, we did not collect data on the type of RTI; a randomised trial of FilmArray® POC testing recorded a shorter duration of antibiotics for patients with asthma and COPD who were in the intervention arm versus the control and a shorter length of stay for COPD patients in the FilmArray® arm [21]. The ED does not routinely use EPMA and so we do not know exactly how many patients received antibiotics there. A negative time to antibiotics, due to administrative errors, was changed to 0 h in 30% of patients however the median was small (−1.5 h) and this phenomenon occurred in both study arms. As in other similar studies on this subject [21] we did not include routine bacteriology results in the analysis however the hypothesis had greater dependence upon the predominantly viral panel results under investigation and bacteriology results would not be expected to differ between the study arms and thus influence results. Finally, the number of eligible patients admitted to the study wards was less than predicted by the data used to plan the study and hence the number of cases recruited fell short of the statistical calculation that required 1131 patients in each arm to detect a fall of 0.6 days. It is not clear why this was the case but may be related to patients by-passing the study wards during the busy winter months of January to March and due to the inclusion of some summer months in the study.

We have selected for patients who required admission and possibly antibiotics by placing the FilmArray® systems on hospital wards. A panel which includes common bacterial causes of lower RTI would probably have identified more pathogens in this setting. A recent study in the UK identified bacteria in lower respiratory tract specimens from 81% of patients with pneumonia [27]. Though we did not record the type of RTI in this study, as in-patients, most patients probably had a lower RTI. A study in the ED might have tested a greater number of patients with a viral illness. There, POC results could provide reassurance that discharge is reasonable. With a rapid result and the broader RP panel afforded by FilmArray®, it is plausible that safety-netting antibiotic prescriptions would have reduced. Due to the mandated maximum 4-h wait for patients in English EDs [30] and a perceived lack of understanding of these results vocalised by our ED staff (because with laboratory-based testing the patient has left ED when results are available), we moved the study one step in to the hospital. This highlights an important knowledge gap. An ED-based study of POC testing incorporating decision making support is therefore advisable.

## Conclusion

We found no association between respiratory multiplex PCR (BioFire FilmArray®) POC testing and length of hospital stay when compared to our routine, laboratory-based respiratory PCR and serology testing. This result was most likely influenced by the delay in the rapid POC testing. POC testing produced results considerably faster than the routine tests but the results were not rapid as designed to be. This was not the fault of the POC test, but highlights the fact that new technology itself is not enough: the correct systems must be in place in order to reap their benefits. Patients who had the POC test received time-critical antivirals for influenza significantly faster and appropriate therapy for mycoplasma infection, not seen in the control arm. Ward staff of all grades performed the POC test without incident meaning that this test has potential across a range of healthcare. Further studies are required that focus on implementing respiratory multiplex PCR POC testing with rapid results, in order to fully assess the impact on length of stay and antibiotic use.

## Abbreviations

AMUs: Acute Medical Units
CAP: Community acquired pneumonia
CFT: Complement fixation test
CRP: C-reactive protein
ED: Emergency Department
EPMA: Electronic patient medicines administration system
EPR: Electronic patient record
EWS: Early warning score
MAC: Medical Assessment Centre
PCR: Polymerase chain reaction
POC: Point of care
RP: Respiratory pathogen
RSV: Respiratory syncytial virus
RTI: Respiratory tract infection
TAT: Turn-around time
WCC: Peripheral white cell count

## References

[1] Surveillance of influenza and other respiratory viruses, including novel respiratory viruses, in the United Kingdom: winter 2012/13, Public Health England. http://webarchive.nationalarchives.gov.uk/20140714084352/http://www.hpa.org.uk/webc/HPAwebFile/HPAweb_C/1317139321787 Accessed on 15 Mar 2017.

[2] Musher DM, Thorner AR (2015). Community-acquired pneumonia. N Engl J Med.

[3] Lim WS, Baudouin SV, George RC, Hill AT, Jamieson C, Le Jeune I, Macfarlane JT, Read RC, Roberts HJ, Levy ML, Wani M, Woodhead MA. Guidelines for the Management of Community Acquired Pneumonia in adults: update 2009. Thorax 2009. Volume 64. Supplement III. 10.1136/thx.2009.121434 19783532

[4] Muthuri SG, Venkatesan S, Myles PR, Leonardi-Bee J, Al Khuwaitir TS, Al Mamun A (2014). PRIDE Consortium Investigators. Effectiveness of neuraminidase inhibitors in reducing mortality in patients admitted to hospital with influenza a H1N1pdm09 virus infection: a meta-analysis of individual participant data. Lancet Respir Med.

[5] WHO Antimicrobial resistance fact sheet http://www.who.int/mediacentre/factsheets/fs194/en/ Accessed on 14 Dec 2016.

[6] Pluddemann A, Onakpoya I, Harrison S, Shinkins B, Tompson A, Davis R, et al. Position paper on anti-microbial resistance diagnostics – Centre for Evidence-Based Medicine. Oxford: University of Oxford June 2015. http://www.cebm.net/wp-content/uploads/2015/07/AMR-Diagnostic-technologies_10-June-2015.pdf Accessed on 15 Mar 2017.

[7] Centers for Disease Control and Prevention. Rapid diagnostic testing for influenza: information for health care professionals. Page last updated: August 4, 2016. http://www.cdc.gov/flu/professionals/diagnosis/rapidclin.htm. Accessed on 8 Sept 2016.

[8] Clark NM, Lynch JP (2011). Influenza: epidemiology, clinical features, therapy, and prevention. Semin Respir Crit Care Med.

[9] Poritz MA, Blaschke AJ, Byington CL, Meyers L, Nilsson K, Jones DE (2011). FilmArray, an automated nested multiplex PCR system for multi-pathogen detection: development and application to respiratory tract infection. PLoS One.

[10] Auburn H, Zuckerman M, Broughton S, Greenough A, Smith M (2011). Detection of nine respiratory RNA viruses using three multiplex RT-PCR assays incorporating a novel RNA internal control transcript. J Virol Methods.

[11] Heim A, Ebnet C, Harste G, Pring-Akerblom P (2003). Rapid and quantitative detection of human adenovirus DNA by real-time PCR. J Med Virol.

[12] CDC protocol of real-time RTPCR for influenza a(H1N1) 28 April 2009 revision 1 (30 April 2009) revision 2 (6 October 2009) http://www.who.int/csr/resources/publications/swineflu/CDCRealtimeRTPCR_SwineH1Assay-2009_20090430.pdf?ua=1 Accessed on 06 Feb 2017.

[13] https://www.cdc.gov/flu/keyfacts.htm last accessed 9 Aug 2017.

[14] Guest JF, Morris A (1997). Community-acquired pneumonia: the annual cost to the National Health Service in the UK. Eur Respir J.

[15] Merchant S, Mullins CD, Shih YC (2003). Factors associated with hospitalization costs for patients with community-acquired pneumonia. Clin Ther.

[16] Royal College of Physicians. National Early Warning Score (NEWS): Standardising the assessment of acute illness severity in the NHS. Report of a working party. London: RCP, 2012. file:///C:/Users/dakshika%20jeyaratnam/Downloads/National%20Early%20Warning%20Score%20(NEWS)%20-%20Standardising%20the%20assessment%20of%20acute-illness%20severity%20in%20the%20NHS_0%20(1).pdf Accessed on 15 March 2017.

[17] Perez K, Olsen RJ, Musick WL, Cernoch PL, Davis JR, Land GA (2013). Integrating rapid pathogen identification and antimicrobial stewardship significantly decreases hospital costs. Arch Pathol Lab Med.

[18] R Core Team (2015). R: a language and environment for statistical computing. R foundation for statistical computing, Vienna, Austria. https://www.R-project.org/.

[19] Van Buuren S, Groothuis-Oudshoorn K (2011). Mice: multivariate imputation by chained equations in R. J Stat Softw.

[20] Wu PJ, Jeyaratnam D, Tosas O, Cooper BS, French GL (2017). Point-of-care universal screening for meticillin-resistant Staphylococcus Aureus: a cluster-randomized cross-over trial. J Hosp Infect.

[21] Brendish NJ, Malachira AK, Armstrong L, Houghton R, Aitken S, Nyimbili E (2017). Routine molecular point-of-care testing for respiratory viruses in adults presenting to hospital with acute respiratory illness (ResPOC): a pragmatic, open-label, randomised controlled trial. Lancet Respir Med.

[22] Xu M, Qin X, Astion ML, Rutledge JC, Simpson J, Jerome KR (2013). Implementation of Filmarray respiratory viral panel in a core laboratory improves testing turnaround time and patient care. Am J Clin Pathol.

[23] Pettit N, Matushek S, Charnot-Katsikas A, Tesic V, Boonlayangoor S, Brielmaier B (2015). Comparison of turnaround time and time to oseltamivir discontinuation between two respiratory viral panel testing methodologies. J Med Microbiol.

[24] Schulert GS, Lu Z, Wingo T, Tang YW, Saville BR, Hain PD (2013). Role of a respiratory viral panel in the clinical management of pediatric inpatients. Pediatr Infect Dis J.

[25] Rogers BB, Shankar P, Jerris RC, Kotzbauer D, Anderson EJ, Watson JR (2015). Impact of a rapid respiratory panel test on patient outcomes. Arch Pathol Lab Med.

[26] Rappo U, Schuetz AN, Jenkins SG, Calfee DP, Walsh TJ, Wells MT (2016). Impact of early detection of respiratory viruses by multiplex PCR assay on clinical outcomes in adult patients. J Clin Microbiol.

[27] Gadsby NJ, Russell CD, McHugh MP, Mark H, Morris AC, Laurenson IF (2016). Comprehensive molecular testing for respiratory pathogens in community-acquired pneumonia. Clin Infect Dis.

[28] Gilbert D, Gelfer G, Wang L, Myers J, Bajema K, Johnston M (2016). The potential of molecular diagnostics and serum procalcitonin levels to change the antibiotic management of community-acquired pneumonia. Diagn Microbiol Infect Dis.

[29] Gelfer G, Leggett J, Myers J, Wang L, Gilbert DN (2015). The clinical impact of the detection of potential etiologic pathogens of community-acquired pneumonia. Diagn Microbiol Infect Dis.

[30] House of Commons Committee of Public Accounts. Department of Health: improving emergency care in England. Sixteenth report of session 2004–05. Report, together with formal minutes, oral and written evidence. Ordered by The House of Commons to be printed 9 March 2005. http://www.publications.parliament.uk/pa/cm200405/cmselect/cmpubacc/445/445.pdf Accessed on 8 Sept 2016.

